# Semiochemical-Mediated Host-Searching and Biological Control Potential of *Trichogramma* Wasps: Mechanisms, Behavioral Plasticity, and Pest Management Applications

**DOI:** 10.3390/plants15121918

**Published:** 2026-06-21

**Authors:** Yu Wang, Xu-Dong Liu, Asim Iqbal, Atif Idrees, Chen Zhang, Wan-Sheng He

**Affiliations:** 1 Agricultural College, Jilin Agricultural Science and Technology College, Jilin 132101, China; wangyu@jlnku.edu.cn (Y.W.); liuxudong20021226@gmail.com (X.-D.L.); 2 Imdaad: Integrated Facilities Management Company, Street Number 1100, South Zone Jebel Ali, Dubai P.O. Box 18220, United Arab Emirates; asim_iqbal990@yahoo.com; 3 Guizhou Provincial Key Laboratory for Agricultural Pest Management of the Mountainous Region, Scientific Observing and Experimental Station of Crop Pest in Guiyang, Ministry of Agriculture and Rural Affairs, Institute of Entomology, Guizhou University, Guiyang 550025, China; atifentomologist@gmail.com

**Keywords:** chemotaxis, host detection, insect behavior, semiochemicals, *Trichogramma*

## Abstract

Globally, *Trichogramma* Westwood (Hymenoptera: Trichogrammatidae) is known as the most effective biological control agent due to its ability to parasitize insect pest eggs. However, identifying an appropriate host is vital for *Trichogramma* to prosper. Therefore, this study delves into the complex role of semiochemicals in shaping the host-seeking behavior of *Trichogramma* parasitoids, with a particular focus on their responses to both plant-derived and host-derived cues. The mechanism of semiochemical reception in *Trichogramma* wasps relies on a highly specialized, sensitive olfactory and gustatory system to locate host eggs and mates. Semiochemicals, which mediate ecological interactions, have been identified as pivotal in influencing the parasitic efficiency of *Trichogramma* species. *Trichogramma*’s host-seeking behavior is influenced not solely by ovipositional cues but also by the intrinsic physical attributes of Lepidopteran hosts, such as the scales on the wings and abdomen, which emit semiochemicals capable of eliciting positive chemotactic responses, thereby guiding parasitoids toward optimal sites for oviposition. Furthermore, the interplay between insect-derived and plant-derived chemical cues exhibits a synergistic effect, collectively enhancing the chemotactic attraction of *Trichogramma*, thereby fine-tuning its host-seeking behavior with greater precision and specificity. This study further underscores *Trichogramma*’s innate behavioral ability to discriminate between host eggs of varying developmental stages, facilitating the precise identification and selection of the most suitable host for parasitization. Age and experience both make *Trichogramma* more selective of hosts, but younger parasitoids may take a broader approach to host selection due to their greater life expectancy. Furthermore, the removal of these cues affects their host localization and learning abilities. Associative learning enables *Trichogramma* to exhibit flexible behaviors, providing them with a selective advantage; allows them to explore various hosts; and reduces environmental uncertainty. Plant structure, host density, and host age are the key factors that significantly influence the foraging and parasitism of *Trichogramma*. The searching speed of this parasitoid is significantly influenced by temperature. Heat stress increases VOC emissions in plants such as potato via stomatal opening, reducing herbivore attraction and enhancing parasitoid recruitment. Furthermore, air pollution, including CO_2_, O_3_, and NOx, impairs parasitoid efficiency by disrupting volatile-mediated host location and reducing biological control performance. *Trichogramma* wasps are generally effective biological control agents, but their success depends on the species used, target pest, crop, release density, and field conditions. Overall, species such as *T. ostriniae*, *T. japonicum*, and *T. leucaniae* show the strongest performance in several crops by increasing parasitism, reducing pest damage, and improving yield. This study highlights the successful integration of semiochemical cues in pest management programs and the effective utilization of *Trichogramma* in conjunction with entomopathogenic bacteria to control Lepidopteran pests. This approach contributes to the development of more effective pest management strategies, thereby promoting agricultural sustainability.

## Simple Summary

*Trichogramma* Westwood (Hymenoptera: Trichogrammatidae) is considered the most effective biological control agent because it parasitizes insect pest eggs. This parasitoid uses plant- and host-derived chemical cues to locate a host and parasitize it. Furthermore, this parasitoid’s innate behavior enables it to precisely identify hosts across various developmental stages. However, *Trichogramma*’s host-seeking ability is disrupted in the absence of semiochemicals. Furthermore, *Trichogramma*’s ability to use associative learning is central to its adaptability and success in pest control.

## 1. Introduction

The search for suitable hosts is a critical aspect of the parasitic behavior exhibited by *Trichogramma* wasps [1,2,3,4,5]. This behavior is governed largely by semiochemicals—volatile organic compounds that facilitate ecological interactions and host detection [6,7,8,9]. The members of the genus *Trichogramma* Westwood (Hymenoptera: Trichogrammatidae) are recognized worldwide as the most promising biological control agents for the sustainable management of agricultural insect pests [10]. *Trichogramma* is the largest genus in the family Trichogrammatidae, with approximately 620 species documented globally [11]. This genus accounts for over one-quarter of the known genera in this family, with the highest numbers of known species in Brazil, China, India, Russia, and the USA [11]. In China, *Trichogramma dendrolimi*, *Trichogramma ostriniae*, *Trichogramma confusum*, *Trichogramma evanescens*, and *Trichogramma japonicum* are the most commonly occurring *Trichogramma* species, exhibiting an overlapping distribution, and they are employed in mass rearing for release against insect pests [11]. Haoxiang, Xiaoqing [12] declared that *T. dendrolimi* and *T. chilonis* are “good regulators” of fall armyworm outbreaks in central and northeastern China. *Trichogramma* parasitoids, which are crucial biological control agents in pest management [13,14], rely on both plant-derived and host-derived chemical cues to locate their hosts [15,16,17]. These cues include a wide range of substances, from plant volatiles induced by herbivore damage to sex pheromones emitted by host insects [18,19,20].

Recent studies have demonstrated that the attraction of *Trichogramma* to these chemical signals is age-dependent, with females at varying stages of maturation exhibiting different levels of sensitivity to these cues [21]. Additionally, the removal of chemical cues from host eggs, such as through rinsing, significantly affects *Trichogramma’s* ability to localize hosts [22]. These age-related and chemical cue-mediated responses highlight the complex relationship between parasitoid behavior and the semiochemicals involved.

As semiochemicals directly impact parasitoid efficiency, understanding these mechanisms offers valuable insights into enhancing pest control strategies. This study investigates the role of chemical cues in *Trichogramma’s* host detection capabilities, aiming to improve our understanding of the ecological dynamics driving their behavior and ultimately contribute to more effective biological pest control methods.

## 2. Review Methodology

A comprehensive literature search was performed running a query comprising keywords and Booleans, namely, (“*Trichogramma*”) AND (“Chemotaxis” OR “Host detection”) AND (“Semiochemicals” OR “Plant-derived cues” OR “Host-derived cues”), across multiple databases, e.g., Google Scholar, PubMed and ScienceDirect. A range of peer-reviewed articles published between 2000 and 2026 was retrieved, and these articles underwent a systematic selection process following the PRISMA guidelines [23].

**Figure 1:**
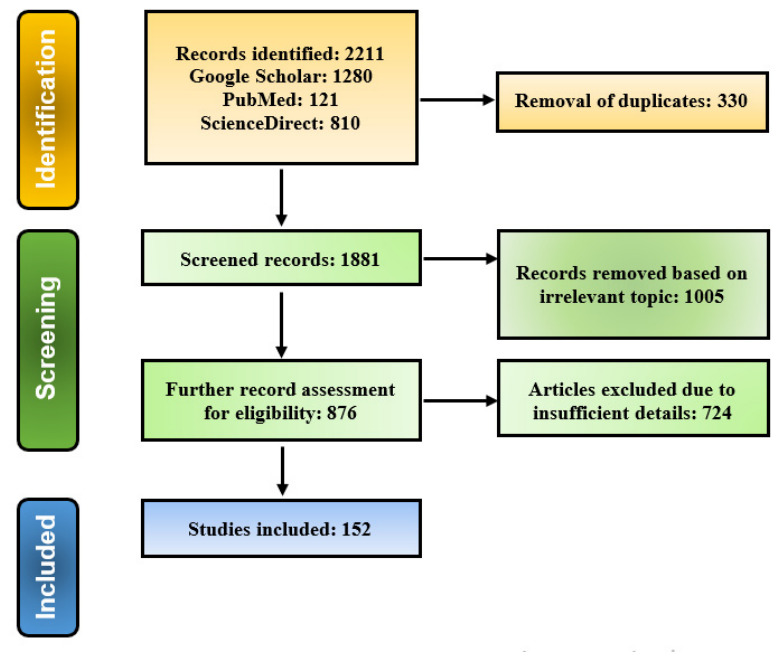
The literature was systematically screened and meticulously retrieved from prominent scientific databases in accordance with the PRISMA guidelines.

**Figure 2:**
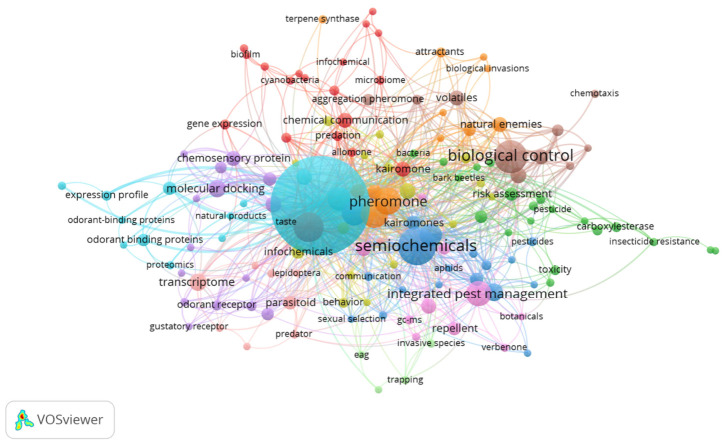
A keyword co-occurrence map illustrating the clustering of frequently used keywords in the literature (generated with VOSviewer version 1.6.19). The size of each node reflects the frequency of keyword occurrence, with larger nodes indicating higher frequency. The connecting lines represent the relationships between the keywords.

**Figure 3:**
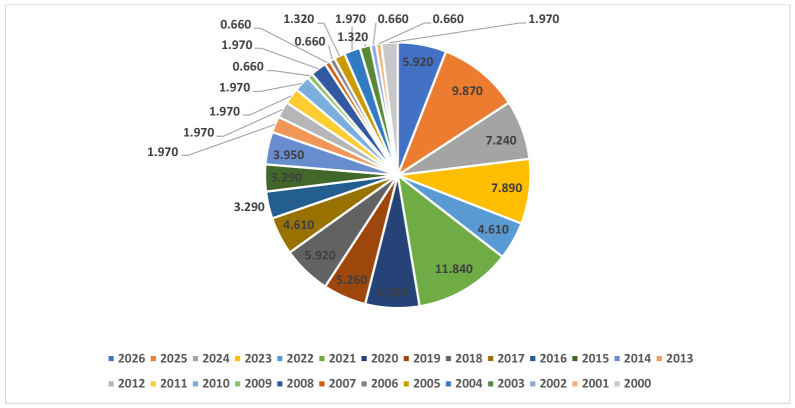
Percent contribution of research articles published in each year.

A total of 2211 records were identified, of which 1881 remained after duplicate removal. Screening based on titles and abstracts resulted in 876 records being retained for further assessment. During the eligibility stage, 724 records were excluded because they did not provide sufficient detail relevant to the objectives of this review. These excluded records mentioned *Trichogramma* only briefly, lacked information on chemotaxis or host-detection behavior, did not report plant-derived or host-derived semiochemical cues, provided no experimental evidence of parasitoid responses to chemical cues, or were conference proceedings and abstracts with insufficient methodological detail. Finally, 152 records met the inclusion criteria and were used for data extraction in this review (Figure 1). The clustering and co-occurrence of the keywords of the selected articles, analyzed using VOSviewer (version 1.6.19), are depicted in Figure 2. A total of 1003 keywords were distributed across nine distinct clusters, with the most frequently occurring keywords being semiochemicals, pheromones, biological control, integrated pest management, kairomones, infochemicals, chemical communication, parasitoid, volatiles, and chemotaxis.Additionally bibliometric parameters included the number of publications in respective years affiliated with the authors of considered articles. About 53.950% of the selected articles were published between 2020 and 2026, indicative of the advancements in the research on this topic (Figure 3). The literature retrieved from the selected databases focused on the semiochemical perception mechanisms in *Trichogramma* wasps, including the impact of chemical cues on chemotaxis, host-searching behavior, and parasitism, as well as the role of plant-derived semiochemicals and environmental factors like plant complexity and host density. Additionally, the illustrations presented in this review were created using BioRender (BioRender.com).

## 3. Mechanisms of Semiochemical Perception in *Trichogramma* Wasps

The mechanism of semiochemical reception in *Trichogramma* wasps relies on a highly specialized, sensitive olfactory and gustatory system to locate host eggs and mates [24]. The complex chemosensory system is composed of multiple chemosensory proteins, including odorant receptors (ORs), gustatory receptors (GRs), and ionotropic receptors (IRs) [25]. A transcriptomic analysis of male and female heads of *T. japonicum* revealed that the differentially expressed genes are primarily associated with chemosensory function. There are sixty-six chemosensory receptor genes, namely, fifty-one ORs, seven IRs, and eight GRs [24]. These genes are more highly expressed in female heads than male heads, likely due to the increased need for females to search for hosts or detect male pheromones [24]. ORs, GRs, and IRs are located in the dendritic membrane of ORNs and are regarded as key factors in the chemosensory signal transduction process [26]. ORs are proteins with a seven-transmembrane domain that function as heterogeneous ion channels composed of a traditional odor receptor (OR) and an odorant receptor co-receptor (ORco). The OR is capable of recognizing odor molecules and pheromones, and it is a highly conserved odorant receptor. However, ORco does not perceive odor molecules and is co-expressed with traditional ORs [27], with which it interacts to form ligand-gated ion channels, enhancing the odor response [28]. GRs exhibit a similar membrane structure to ORs but are distantly related to ORs in the G-protein-coupled receptor family and are principally expressed in gustatory receptor neurons [29]. IRs are a specialized, ancient subfamily of ligand-gated ion channels, divergent from inotropic glutamate receptors (iGluRs), that function primarily as chemosensory receptors in insects [30]. Zhang, Jiang [31] identified one-hundred ORs, twenty-seven GRs, and twenty-seven IRs in the genome of *T. dendrolimi*.

## 4. Pheromone and Egg Surface Volatiles as Semiochemical Signals Guiding *Trichogramma* Host Detection

Semiochemicals constitute a diverse class of signaling chemicals that mediate interactions between living organisms, directing their behavior and making changes within them [32]. These chemicals, produced by plants, insects, or microbes, convey critical information that influences insect movement, aggregation, mating, oviposition, and foraging activities [33]. Insects, particularly those in the Lepidoptera (butterflies and moths) and Coleoptera (beetles) orders, secrete a vast array of semiochemicals, with their sex pheromones and aggregation pheromones being the most extensively studied and utilized for communication [34,35]. Ouaba, Tchuinkam [36] declared that Lepidoptera is the second most diverse pest insect order after Coleoptera, with more than 178,159 species described in more than 4000 genera worldwide. Lepidopteran pests cause damage to cultivated crops and other production systems, resulting in significant economic losses, mainly pronounced in cereal crops (millet, maize, rice, and sorghum), horticultural crops (cabbage, green beans, sweet potatoes, and tomatoes), and honey production in Cameroon [36]. During a previous light trap study conducted in China [37], the majority of insect pests captured were from the order Lepidoptera, highlighting their dominance in nocturnal pest populations and the importance of targeting these groups in pest management strategies. Semiochemicals are behavior-modifying compounds, including pheromones, that can attract or disrupt the mating of insects, allowing them to communicate in ways invisible to humans [38].

*Spodoptera frugiperda* Smith (Lepidoptera: Noctuidae) is a highly destructive polyphagous pest of more than 350 plant species from at least 76 plant families worldwide [39,40]. The female moth lays 100–180 dome-shaped eggs in clusters on leaves, secured with adhesive secretions and covered with protective barbed hairs [41]. These protective substances on their eggs have been recorded as semiochemicals, such as kairomones for *Trichogramma*, which influence the chemotactic behavior of this parasitoid [42]. The scales on *S. frugiperda* eggs, wings, and abdomen release volatiles, including acetates, alcohols, aldehydes, carboxylic acids, hydrocarbons, and ketones [42]. However, *Trichogramma pretiosum* actively attracts two acetate compounds, (Z)-9-tetradecenyl acetate (Z9-14:OAC) and (Z)-11-hexadecenyl acetate (Z11-16:OAC), compared to others. Furthermore, *T. pretiosum* exhibits positive chemotaxis that varies with pheromone concentration in such a way that, in a previous study, it was found that wasps could recognize Z9-14:OAC at all three tested concentrations (1, 0.1, and 0.01 µg µL^−1^), while Z11-16:OAC was attractive only at very low levels (0.1 and 0.01 µg µL^−1^). This difference was attributed to olfactory binding proteins (OBPs) in the antennal sensilla [43], which are responsible for intercepting chemical molecules and transducing this signal to the sensilla nerve endings [44]. A higher number of OBPs tuned to Z9-14:OAC likely enables consistent detection across a wide range of doses. In contrast, fewer OBPs are capable of recognizing Z11-16:OAC. Consequently, higher concentrations of this compound can saturate these proteins, preventing effective signal transmission and resulting in the observed lack of response. The results show that the chemotactic behavior of *Trichogramma* is shaped by the specificity of OBPs to certain semiochemicals, influencing host detection efficiency. Recently, Kong, Zhang [45] also identified a key OBP that is highly expressed in the female head of *T. ostriniae* and exhibits high and broad affinity for both host sex pheromones ((Z)-12:14AC and (E)-12:14AC) and egg surface volatiles. Future studies should focus on identifying these OBPs and their interactions. Additionally, RNAi-mediated silencing of OBPs could be applied to enhance biological control strategies using semiochemical-based tools.

## 5. Plant-Derived Volatile Cues in *Trichogramma* Host Location and Egg Selection

**Figure 4:**
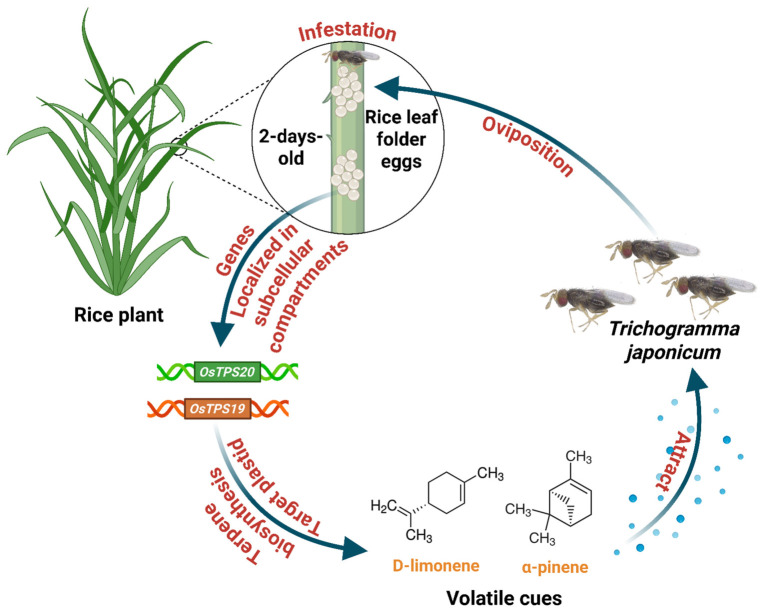
Interaction between rice plants, rice leaf folder eggs, and *Trichogramma* japonicum. Created in BioRender. Ahmed, K. S. (2026) https://BioRender.com/vcvg1cf (accessed on 3 June 2026).

Semiochemicals play a crucial role in shaping the host-seeking behavior of *Trichogramma* egg parasitoids [22]. These chemical cues influence the various stages of the host utilization process, including habitat location, host location, and host acceptance [46]. In *Trichogramma*, host-search behavior is guided by well-established semiochemical cues, including both plant volatiles and host-derived chemicals [9]. Among these, herbivore-induced plant volatiles (HIPVs) have been shown to be particularly effective in attracting parasitoids to suitable hosts. For instance, *Trichogramma achaeae* females are innately attracted to the volatiles produced by tomato plants, regardless of infestation status [9]. However, oviposition and larval feeding by the pest *Tuta absoluta* significantly enhance HIPV emission, potentially improving parasitoid attraction [9]. Host sex pheromones can also influence *Trichogramma* behavior, as demonstrated by *T. achaeae*’s attraction to *T. absoluta*’s sex pheromones [9]. In addition to semiochemicals alone, the blends of volatiles also provide cues in *Trichogramma* host-seeking in such a way that anisole and bis(2-ethylhexyl) phthalate volatiles emitted by host eggs (*Helicoverpa zea*) and infested plants (*Crotalaria juncea* infested with *H. zea*), respectively, synergistically attract *Trichogramma papilionis* even at long distances, ultimately optimizing and improving their search strategy [47]. A recent study [48] also rejected the traditional thought that *Trichogramma* only uses contact cues, i.e., direct physical or chemical interaction with host eggs, to assess their quality. The authors found that wasps assess host egg quality from a distance, using volatile cues emitted by the plants on which the eggs are carried. An olfactometer assay was conducted to assess the preference of *T. japonicum* for plant volatiles emitted by rice plants bearing eggs of different ages (1–4 days) of the rice leaf folder *Cnaphalocrocis medinalis*, compared with volatiles from uninfested rice plants. [48]. The results indicated that wasps were significantly more attracted by the odor (D-limonene and α-pinene volatiles) of the rice plants carrying the 2-day-old eggs of *C. medinalis* [48]. This is because the genes *OsTPS19* and *OsTPS20*, which are localized in subcellular compartments in rice tissues, play a significant role in targeting plastids for terpene biosynthesis, with the highest expression observed in plants with 2-day-old embryos (Figure 4) [48]. Furthermore, rice plants without eggs, as well as those with eggs of varying ages, were not particularly appealing to the female *Trichogramma* due to a decreased amount of terpenes released by both the host and the plants [48]. *T. japonicum* exhibits an innate ability to discriminate among host eggs by exploiting plant-derived volatiles, particularly α-pinene and D-limonene, which reliably signal the presence and suitability of 2-day-old eggs [48]. This strategy allows the wasp to optimize offspring performance while minimizing foraging costs, reflecting a finely tuned evolutionary adaptation with direct implications for sustainable pest management.

## 6. Plant-Derived Semiochemicals and Their Impact on *Trichogramma* Behavior

Plant cues, particularly volatile organic compounds, are vital mediators of ecological interactions, enabling plants to anticipate threats, fine-tune adaptive responses, and optimize survival. Their specificity and context-dependency not only enhance individual fitness but also shape community dynamics and trophic interactions [49]. Parasitic organisms, particularly parasitoid females, are remarkably skilled in utilizing many plant-derived cues to locate their hosts [17], as olfactory cues play a pivotal role in host location and egg disposition in parasitoids [50]. Plants release their cues into the environment through the medium roots or leaves as a response to oviposition [51,52,53]. In plants, responses are stimulated through chemicals present in herbivore regurgitation [54], oviduct secretion, and the oral secretion of some larval species of Lepidoptera [54,55,56], which act as elicitors. Afentoulis, Cusumano [57] found a plant, the wild crucifer *Brassica nigra*, in a tight spot due to oviposition by a herbivorous insect pest, the cabbage white butterfly (*Pieris* sp.). As a response, oviposition-induced plant volatiles (OIPVs), such as (E)-caryophyllene (a sesquiterpene), attracted *T. evanescens*. In addition to OVIPs, plants release herbivore-induced plant volatiles (HIPVs) in reaction to herbivore attacks, which they use to recruit tritrophic natural enemies, acting as a “cry for help” by signaling herbivores to parasitoids that prey on them [58]. Moreover, the application of promising herbivore-induced plant volatiles (HIPVs) in controlled-release formulations under field conditions can act as arrestants, effectively attracting *Trichogramma* populations and increasing their residence time while searching for hosts at different developmental stages, thereby enhancing crop pest management. [58]. Octadecane, an HIPV, was used in the formulation of kairomone gel, and its efficacy on the biocontrol potential of *T. chilonis* against the wheat pink stem borer *Sesamia inferens* and the chickpea pod borer *Helicoverpa armigera* was examined under field conditions [58]. The gel formulation of octadecane, when applied 24 h after releasing *T. chilonis*, enhanced its foraging activity by acting as a kairomone to attract parasitoids to pest eggs, ultimately reducing dead hearts by up to 48.53% in wheat and pod damage by 31.22% in chickpea [58]. More studies on the effects of plant-derived semiochemicals on *Trichogramma* behavior are described in Table 1.

## 7. *Trichogramma*’s Detection of Plant Hypersensitive Response to Herbivore Eggs

**Figure 5:**
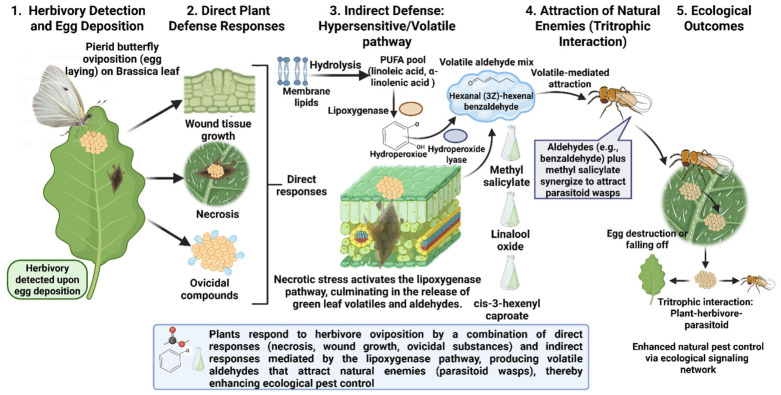
Plants respond to herbivore oviposition through a combination of direct responses (necrosis, wound growth, ovicidal substances) and indirect responses mediated by the lipoxygenase pathway, producing volatile aldehydes that attract natural enemies (parasitoid wasps), thereby enhancing ecological pest control. Created in BioRender. Ahmed, K. S. (2026) https://BioRender.com/phrg105 (accessed on 3 June 2026).

Plants have the potential to detect herbivory soon after herbivores oviposit their eggs on plant parts and initiate a range of direct and indirect defense responses to reduce the survival of the herbivores’ eggs (Figure 5). The direct reactions include wound tissue growth, the formation of necrotic cells, and the production of ovicidal substances [72,73]. Additionally, Griese, Caarls [74] identified an indirect response in *Brassica* crops: they express a hypersensitive response, such as necrosis, underneath pierid butterfly eggs, which leads to the eggs desiccating or falling off the plant. Under necrotic stress, plants activate the lipoxygenase pathway, which involves the hydrolysis of membrane lipids, releasing polyunsaturated fatty acids (PUFAs) such as linoleic acid and α-linolenic acid [75] (Figure 5). These PUFAs are oxygenated by lipoxygenase enzymes, resulting in the production of hydroperoxides. The hydroperoxide lyase enzyme then cleaves these hydroperoxides into volatile aldehydes (e.g., hexanal, (3Z)-hexenal), which are key components of green leaf volatiles [75]. Aldehydes, such as benzaldehyde, play a crucial role in attracting *T. dendrolimi* to orchards. When combined with methyl salicylate and supplemented with linalool oxide or cis-3-hexenyl caproate, these formulations significantly outperformed the control (methyl salicylate), demonstrating the key role of aldehydes in enhancing parasitoid attraction [61,76]. By emitting these volatiles, plants not only defend themselves but also trigger an ecological signaling network, enhancing tritrophic interactions (plant–herbivore–natural enemy) and promoting natural pest control [76] (Figure 5).

## 8. Age- and Experience-Dependent Responses of *Trichogramma* to Chemical Cues

### 8.1. Changes in Chemical Sensitivity with Age

Parasitoid age is the most important factor influencing the sensitivity of *Trichogramma* to semiochemicals [77]. Guazzelli, Giustina [22] stated that age plays a significant role in *T. pretiosum*’s chemotactic response and parasitism efficiency. Furthermore, the authors employed the synthetic sex pheromone of the oriental fruit moth *Grapholita molesta* (Busck, 1916), which consists of the components Z-8-dodecenyl acetate, E-8-dodecenyl acetate, and Z-8-dodecenol in a ratio of 93:6:1, to assess the olfactory response of *T. pretiosum*. The results revealed that mated male *T. pretiosum* did not rely on the pheromone for host-searching. However, mated females aged 24–72 h were attracted to the pheromone blend, while 96-h-old females showed a preference for hexane, suggesting an age-related decline in olfactory capabilities. Moreover, experience enhances host recognition, as evidenced by the observation that inexperienced *T. pretiosum* females exhibit higher parasitism rates in rinsed eggs (*G. molesta* eggs immersed in a solvent to remove surface chemicals) at 72 and 96 h. However, with age, the females shift their preference to unrinsed eggs, indicating that experience improves their ability to recognize the host [22].

### 8.2. Learning and Memory in Trichogramma’s Host-Searching Behavior

Research into the effects of miniaturization on the cognitive abilities of insects is indeed one of the most promising and active fields in neurobiology [78,79,80]. Fedorova, Farisenkov [81] investigated the cognitive abilities of *T. telengai* (Sorokina, 1987), focusing on associative learning and memory despite their small size. Using a thermal arena based on the Morris water maze, the study trained the insects to find a “comfort zone (cooler area)” and tested memory retention up to six hours post-training. The results showed significant memory retention, with the test group spending more time in the target sector (comfort zone) and demonstrating shorter travel paths after repeated training, indicating improved learning. The learning index also revealed significant differences between the test and control groups, demonstrating the insect’s ability to learn and retain information.

## 9. Role of Experience and Chemical Cues in Host Selection Behavior of *Trichogramma*

Host insect-derived cues play a crucial role in guiding the foraging behavior of *Trichogramma*, enabling it to effectively locate its hosts. Acetophenone, anisole, β-myrcene, bis(2-ethylhexyl) phthalate, benzene, (1-methylethyl), and nonanal are chemical cues secreted from *H. zea* eggs perceived by *T. papilionis* for parasitism [47]. Based on the identification of these volatiles in host eggs, the following questions arise: firstly, is *Trichogramma’s* attraction to insect-derived cues age-dependent, and how might this affect its efficacy in biological pest control? Secondly, how does the removal of chemical cues from host eggs (e.g., through rinsing) affect the learning and host localization behavior of *Trichogramma* parasitoids?

**Figure 6:**
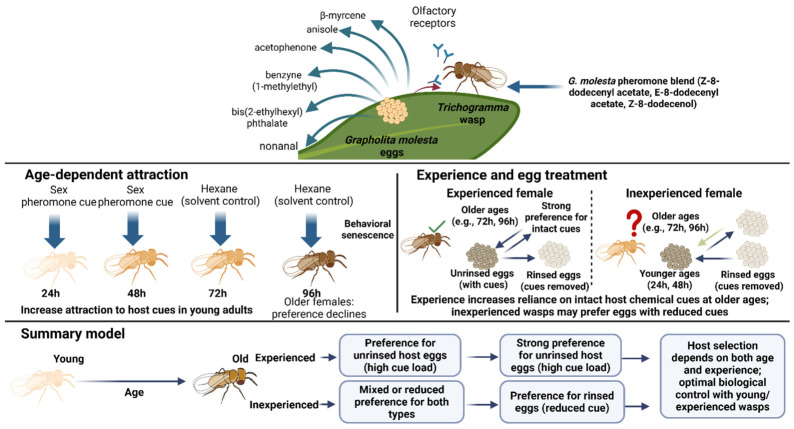
Age and experience dependence of *Trichogramma* host location behavior via insect-derived chemical cues. Created in BioRender. Ahmed, K. S. (2026) https://BioRender.com/zmxo305 (accessed on 3 June 2026).

While seeking answers to these questions, we found a recent study conducted by Guazzelli, Giustina [22], who utilized a blend of sex pheromones from *Grapholita molesta*, including Z-8-dodecenyl acetate, E-8-dodecenyl acetate, and Z-8-dodecenol (93:6:1) [82]. Sexually mature (mated) *T. pretiosum* males and females of varying ages (24, 48, 72, and 96 h old) were exposed to the pheromones. The result indicated that mated females (24, 48, and 72 h old) were more attracted to the sex pheromone blend of *G. molesta* than to hexane (control) [22]. In contrast, when female wasps were 96 h old, they preferred the solvent over the pheromone blend [22], indicating that aging leads to behavioral senescence, where both olfactory sensitivity and exploratory activity decline significantly [83]. Ahmadi and Poorjavad [21] also found that the attractiveness of *T. evanescens* females to *T. absoluta*’s sex pheromone is impacted by age, with younger females responding more strongly than older females. When evaluating the effectiveness of the parasitoid, experience is a far more important aspect than the age of *Trichogramma*. In a previous study, experienced (previously encountered the host egg) and inexperienced (had never encountered the host egg) *T. pretiosum* females of varying ages were exposed to rinsed (washed with a solvent, hexane) and unrinsed eggs of *G. molesta*. The results revealed that experienced wasps showed a higher parasitism rate in unrinsed eggs at older age (72 and 96 h) (Figure 6). In contrast, inexperienced wasps preferred rinsed eggs at these ages, suggesting that older parasitoids, particularly those with prior experience, rely more on chemical cues to identify suitable hosts. At the same time, younger parasitoids may exhibit a broader approach to host selection due to their longer life expectancy. Nevertheless, additional experiments are required to comprehensively assess the comparative preference for various host types and to substantiate the occurrence of learned behavior in inexperienced female parasitoids (Figure 6). Such studies will be essential to validate the extent to which experience influences host selection and to confirm the mechanisms underlying this learned behavior in naïve individuals.

## 10. Effect of Associative Learning on Host-Searching Behavior

The host-searching behavior of parasitoids is influenced by associative learning [84]. Associative learning is a cognitive process typically associated with the use of chemical cues during host-searching by parasitoids. This ability enables parasitoids to exhibit flexible behaviors, providing them with a selective advantage, and it allows them to explore various hosts and reduces environmental uncertainty [85]. In this context, Gonthier, Romeis [86] demonstrated that the parasitoids *T. achaeae* and *T. evanescens* can learn to associate tomato odor with *T. absouluta* eggs, enhancing their host-searching efficiency. These results were demonstrated through olfactometer assays, where conditioned parasitoids spent more time in chambers containing the odor of infested tomato leaves than naive parasitoids, who exhibited a repellent response. This behavioral flexibility provides a selective advantage, as it allows parasitoids to explore new hosts such as tomato plants that they might otherwise avoid, thereby improving their parasitism rates [86].

## 11. Interactions Between Plant Structure, Host Density, and Searching Speed

### 11.1. Effect of Plant Complexity on Host-Searching Efficiency

The complexity of the plant structure significantly affects the host-finding efficiency of *Trichogramma* parasitoids [87]. Gingras, Dutilleul [88] stated that there was no significant plant preference between the two parasitoid species *T. evanescens* and *T. pretiosum*, although parasitism rates were highest on cabbage and lowest on Brussels sprouts. Parasitism decreased with increased plant age and structural complexity, while cabbage had the highest parasitism rate due to its simpler structure. *T. evanescens* outperformed *T. pretiosum* across most plant varieties. Additionally, parasitism was higher on the abaxial leaf surface and at the base of the plant, particularly in older plants [88]. Gingras, Dutilleul [89] also found that simple plant structures, characterized by fewer leaves, less complexity, and fewer connections between plant parts, enabled *Trichogramm turkestanica* Meyer to search more efficiently for eggs of the Mediterranean flour moth *Ephestia kuehniella* (Zeller, 1879). Furthermore, on complex plant structures, *T. turkestanica* spent more time without encountering eggs and less time actively exploring.

### 11.2. Host Density and Its Influence on Foraging Success

Host egg density plays a crucial role in the parasitism success of *Trichogramma*. A higher host egg density improves foraging success and increases offspring emergence for parasitoids [90]. Furthermore, the density of one-hundred eggs of the rice moth *Corcyra cephalonica* provides more resources for oviposition and significantly improves offspring emergence rates for both *T. ostriniae* and *T. dendrolimi*. At lower host densities (10, 20, and 30 eggs), competition for limited resources is stronger, and emergence rates vary more widely depending on the parasitoid species and oviposition sequence, indicating that, at lower host densities, foraging success may be reduced because the parasitoids compete more intensely for fewer hosts [90].

### 11.3. Influence of Host Age on Parasitism by Trichogramma Parasitoids

Host age is also a key factor that influences the parasitism of the parasitoid [91,92]. In this context, Sun, Yan [93] revealed that host egg age significantly influences the parasitism behavior and fitness of *T. chilonis*. Furthermore, *T. chilonis* performs optimally on younger eggs (24–72 h) of the potato tuber moth (*Phthorimae operculella* Zeller, 1873), which provide better nutritional value and support higher offspring emergence rates [93]. Parasitizing older eggs (96+ hours) results in reduced offspring fitness, a smaller body size, and lower fecundity due to the depletion of nutrients and accumulation of defensive substances in older eggs [93,94]. Queiroz, Costa [95] also suggested that the parasitism rate of *T. pretiosum* is strongly influenced by host egg age, with the highest parasitism occurring in 24-h-old eggs of the velvetbean caterpillar *Anticarsia gemmatalis* (Hübner, 1818).. These findings are crucial for optimizing the release of *T. pretiosum* in field application. To maximize the efficiency of biological control, it is important to synchronize the release of *T. pretiosum* with the most susceptible stage of the host, which is the 1-day-old egg. *T. chilonis*, *T. dendrolimi*, *T. japonicum*, and *T. ostriniae* perform well on 1-, 2-, and 3-day-old eggs of the rice leafroller *Cnaphalocrocis medinalis* (Guenee, 1854) [96]. Jiang, Zhou [97] stated that *T. ostriniae* is one of the most promising candidates for advancing biological control strategies against the tomato leafminer *Tuta absoluta* (Meyrick, 1917), as this *Trichogramma* species effectively parasitizes *T. absoluta* eggs at all developmental stages, positioning it as a potential dominant parasitoid for controlling *T. absoluta* in China.

### 11.4. Searching Speed and Host-Finding Behavior in Trichogramma

The searching speed of *Trichogramma* is the key factor for measuring parasitoid quality [98]. Two- and three-day-old *T. minutum* searched twice as fast as one-day-old females due to physiological development, increased reproductive motivation, refined foraging skills, and enhanced energy reserves [98]. Suverkropp, Bigler [99] also described the searching pattern of *Trichogramma* species on maize plants at varying temperatures (18 °C and 25 °C) in relation to residence time. Residence time is also a crucial determinant of host-finding behavior in parasitoids [100]. *T. brassicae* parasitoids spent an average of 44.9 min on a maize plant at a cooler temperature of 18 °C, compared to 20.8 min at a warmer temperature of 25 °C. Furthermore, the parasitoid primarily moved from one leaf to another and showed a clear preference for leaf areas closest to the stem [99].

## 12. Environmental and Climate Change Effects on Semiochemical Signaling

### 12.1. Impact of Climate Change on Volatile Organic Compound (VOC) Emissions and Parasitic Behavior

Climate change alters the emission patterns of volatile organic compounds (VOCs) from plants, which in turn influences tritrophic interactions, including those between plants, herbivores, and their natural enemies such as parasitoids [101,102]. VOCs are a class of biogenic chemicals released by plants, and they serve as infochemicals [103] that mediate communication within ecological networks and guide insect behaviors [104,105] like food location, host foraging, mate-finding, and parasitism [106]. VOC induction in plants after exposure to heat [107], drought [108], and elevated ozone (O_3_) levels [109] may increase parasitoid attraction to plants in the absence of herbivores. Munawar, Zhang [107] revealed that high-temperature treatment of potato plants increased the emission of VOCs, notably β-caryophyllene, which played a critical role in plant defense by deterring the herbivore *P. operculella* while attracting the parasitoid *T. chilonis* and repelling herbivores. Furthermore, the heat stress enlarged stomatal apertures, increasing VOC release from potato leaf tissues [107].

### 12.2. Impact of Air Pollution on Trichogramma

Air pollution is an increasing global concern, presenting significant risks to both human health and the environment [110,111]. Furthermore, greenhouse gas pollutants such as carbon dioxide and air pollutants such as O_3_ or diesel exhaust represent major threats to economically important insects, particularly pollinators and natural pest regulators [112,113]. CO_2_ decreases the performance and parasitism rate of *T. pretiosum* [114]. Furthermore, air pollutants, particularly O_3_ and nitrogen oxides (NO_x_) from diesel exhaust, significantly disrupt the olfactory-guided foraging behavior of parasitoids by degrading host-related plant volatiles and reducing the insect’s ability to detect chemical signals [115].

## 13. Novel Approaches for Enhancing Biological Control Through Chemical Cues

### 13.1. Integrating Semiochemical Technology into Pest Management

The integration of semiochemical technology into pest management has led to several innovations, including enhancing the searching efficiency of natural enemies, directing natural enemies into a specific search mode, making novel or artificial host–prey species suitable for mass rearing, and breeding plant cultivars that emit more natural enemy-attracting synomones [116]. The “push–pull” strategy in pest management utilizes infochemicals to influence insect pest behavior. Insect pests are repelled from crops using chemical cues, such as (E)-β-ocimene and α-terpinolene, which obscure host plant signals. Concurrently, pests are attracted to traps or trap crops using attractive stimuli, facilitating their concentration for more effective control. This innovative strategy enhances integrated pest management by combining repellents and attractants for improved pest control efficiency [116]. Rani and Murali-Baskaran [117] demonstrated that several synthetic phenolic compounds, which are common plant secondary metabolites involved in herbivore-induced defense, function as semiochemical cues for *T. chilonis*. These compounds were applied directly in laboratory bioassays, including culture tube assays, Y-tube olfactometer assays, host egg surface treatments, and artificial plant models, to evaluate parasitoid orientation and parasitization responses. The study showed that *T. chilonis* females were attracted to specific phenolics, particularly syringic acid, quercetin, coumaric acid, pyrocatechol, and chlorogenic acid, and that some treated host eggs showed enhanced parasitization. These findings suggest that phenolic compounds can act as natural plant-derived signals or synomones that help egg parasitoids locate host-associated sites, thereby supporting their potential use in biological control strategies. In contrast, *Trichogramma cordubensis* females were reported to be attracted to honey and the sex pheromone of the armyworm moth *Mythimna unipuncta* but repelled by vinegar and peppermint essential oil [118], indicating that both attractive and repellent semiochemicals may be incorporated into pest management strategies.

### 13.2. Developing Multi-Agent Pest Control Strategies

The integration of more than one control method is the most promising tactic to control insect pests [119]. The use of the parasitoid wasps *Trichogramma* spp. and the bioinsecticide *Bacillus thuringiensis* to improve biological control is among the common alternative components of integrated management of various insect pests [120]. Nascimento, Fadini [121] investigated the interaction between two biological control agents, *T. pretiosum* and *B. thuringiensis*, and found that this entomopathogen did not negatively affect the parasitoid’s behavior in selecting pest eggs, highlighting that the combined use of these agents offers an effective approach to control insect pests. Furthermore, in Iran [122,123] and China [124], various *Trichogramma* species have been utilized in combination with *B. thuringiensis* to significantly control various Lepidopteran pests in both greenhouses and fields.

## 14. Effects of Host Egg Quality on Parasitism Behavior and Parasitoid Performance

### Role of Physical Egg Quality in Parasitism

Host egg quality plays a critical role in shaping parasitism behavior and parasitoid performance [125]. Du, Xu [126] demonstrated that *Trichogramma* parasitoids, including *T. japonicum*, *T. chilonis*, and *T. leucaniae*, parasitized significantly more fertilized eggs than unfertilized ones. Furthermore, the parasitoids developed faster on fertilized eggs than unfertilized eggs. Regarding ovipositional behavior, parasitoids rejected unfertilized eggs after drumming about 3.7 times more often than fertilized eggs. Giri, Pokhrel [127] highlighted the influence of the physical quality of the eggs of the fall armyworm *S. frugiperda* (J.E. Smith, 1797), including the scale coverage. The result indicated that *T. chilonis* encountered a physical barrier and faced difficulty parasitizing fully covered egg masses compared to partially covered or uncovered eggs.

## 15. Applications of Biological Control with *Trichogramma* Wasps

Biological control using *Trichogramma* wasps is a highly effective and sustainable method for pest management, particularly for controlling Lepidopteran pests in agricultural systems [4,11,128,129]. One of the most successful applications of *Trichogramma* is in controlling the Asian corn borer *Ostrinia furnacalis*, a major pest in maize crops. In northeastern China, the area of maize treated with *Trichogramma* release for corn borer control increased from 600,000 to 5,500,000 hectares between 2005 and 2015, representing 35% of the total corn cultivation area in this key corn-producing region [130,131]. To optimize biological control programs, it is crucial to identify the optimal number and developmental stage of natural enemies to be released. In this context, Wang, Hou [132] assessed the parasitism abilities of *T. dendrolimi* and *T. ostriniae* under two release methods: the same developmental stage (SDS) and different developmental stages (DDSs). A total of 10,000 wasps from both species were released using these methods. The results showed that *T. dendrolimi* achieved higher parasitism rates and longer effectiveness (6 days) with the DDS method than the SDS method (3 days). However, the release method had no impact on *T. ostriniae*, which effectively parasitized *O. furnacalis eggs* in both methods. The study suggests that the DDS method improves the field performance of *T. dendrolimi*. More studies on the use of *Trichogrmma* species as biocontrol agents are described in Table 2.

## 16. Non-Chemical Cues and Their Role in Host-Searching Behavior

### Visual Cues in Host Location

Chemical cues, such as plant or host volatiles, have been extensively documented in host location studies [48,143]. However, visual cues are progressively being acknowledged for their significant role, particularly in short-range host-searching behaviors [144]. The *T. ostriniae* species exhibited a distinct preference for specific egg colors during its short-range searching behavior. When using colored clay beads to simulate host eggs, the wasps demonstrated a clear preference for yellow over other colors, ranking them as follows: white > yellow > green > black. This preference corresponds to the visual characteristics of the European corn borer’s eggs, the wasp’s primary target [5]. Furthermore, wasps likely utilize color to assess the suitability of potential hosts, with visual cues acting as an efficient means of discriminating between viable and non-viable eggs. Notably, black-colored beads, which may resemble parasitized or damaged eggs, were strongly rejected by the wasps [5]. Keasar, Ney-Nifle [145] demonstrated that *Trichogramma thalense* exhibits associative learning, where visual cues are shaped by prior experiences. Wasps exposed to black egg cards with *A. kuehniella* hosts preferred the black background in subsequent tests, while those exposed to green egg cards with hosts preferred the green background. These results suggest that *T. thalense* forms visual memories associated with host presence, which influences its foraging behavior and enhances host selection efficiency in subsequent phases. These findings challenge the view that parasitoids rely solely on chemical cues and highlight their capacity to form and retain visual memories. This ability could improve biological control programs, as visual cues could be utilized for targeted pest management, thereby opening new research avenues into parasitoid cognition and ecological applications [145]. Furthermore, *Trichogramma* possesses a limited sensory detection range due to its very small body size and restricted perceptual capacity. Therefore, during short-range host-finding, visual cues may provide more immediate, precise, and reliable information than olfactory cues. When host eggs are visually conspicuous in terms of size, color, shape, or contrast with the surrounding surface, the parasitoid can detect and approach them more effectively through visual orientation. In contrast, olfactory cues released from host eggs may be weak, rapidly dispersed, or difficult to distinguish from background environmental odors at very small spatial scales. As a result, odor alone may not consistently guide the wasps toward suitable hosts. However, when eggs are large and visually prominent, such as *Manduca sexta* eggs, olfactory signals can complement visual cues by creating a stronger multimodal stimulus. This combined visual and chemical information may enhance host recognition and improve host-finding efficiency. Overall, the findings suggest that short-range host location in *Trichogramma* is primarily influenced by visual perception and random movement, while odor plays a supplementary role under specific conditions where it is associated with clear visual signals [146].

## 17. Thioredoxin System

### 17.1. Role of Thioredoxin (Trx) in Insect Physiology

The thioredoxin (Trx) system is a critical component of an insect’s antioxidant defense system [147]. This system, composed of thioredoxins (Trxs) and thioredoxin reductase (TrxR), helps regulate the cellular redox state by reducing oxidized proteins and protecting cells from oxidative stress [148]. Oxidative stress arises from an imbalance between the production of reactive oxygen species (ROS) and the body’s ability to neutralize them, which can lead to the damage of proteins, lipids, and DNA [148]. In insects, the Trx system plays an essential role in maintaining cellular function, particularly in response to environmental stressors like temperature fluctuations [149] and pesticide exposure [150]. Furthermore, during insect diapause, the Trx genes AcTrx2 and AcTrx-like play a key role in antioxidant defense by maintain redox balance, with knockdown triggering compensatory antioxidant responses. A study investigated the thioredoxin genes AcTrx2 and AcTrx-like in *Arma chinensis* (a predatory stink bug) diapause, revealing their upregulation under oxidative stress to maintain redox balance. Knockdown of these genes increased ROS levels and activated other antioxidant enzymes like superoxide dismutase (SOD) and catalase (CAT), showing their central role in managing oxidative stress [151]. However, direct measurement of thioredoxin (Trx) activity in *Trichogramma* during host searching is necessary to further clarify its role in regulating oxidative stress. Additionally, exploring its interaction with enzymes like SOD and CAT could reveal strategies to enhance survival and biological control efficiency.

### 17.2. Trx and Its Antioxidant Functions

The antioxidant properties of Trx are primarily due to its ability to donate electrons to other proteins in the cell [152]. Trx reduces disulfide bonds in target proteins, converting them back to their active, reduced states. This ability is crucial for maintaining the functionality of metabolic enzymes, transcription factors, and components of the immune system in insects [148]. In addition to its antioxidant properties, Trx is involved in regulating various biological processes, including cell proliferation, apoptosis, and immune responses [148]. Trx and thioredoxin reductase (TrxR) also help regulate other antioxidant enzymes, such as peroxiredoxins (Prxs) and methionine sulphoxide reductases (Msrs), which are important in detoxifying ROS [148]. In insects, this process ensures that oxidative damage is minimized, allowing the insects to maintain their health and reproductive potential, which are key to their success in biological pest control.

## 18. Conclusions and Future Perspective

The findings of this study highlight the crucial role of semiochemicals in influencing the host-seeking behavior of *Trichogramma* parasitoids. Both plant-derived and host-derived chemical cues significantly influence the parasitoids’ ability to locate hosts, with particular emphasis on age-dependent responses. The study also highlights the profound impact of chemical cue removal on *Trichogramma*’s host localization, suggesting that chemical cues play a vital role in learning and host detection processes. These insights into the semiochemical-driven behavior of *Trichogramma* provide a deeper understanding of its potential in integrated pest management. Future research should focus on identifying the specific plant- and host-derived cues that trigger the strongest responses from *Trichogramma* parasitoids, particularly in different environmental contexts.

## Figures and Tables

**Table 1:** Effect of plant-derived semiochemicals on *Trichogramma* behavior.

Effect on *Trichogramma* Behavior	*Trichogramma* Species	Chemical Class	Identified Compound(s)	Source Plant	Reference
Attractive	*Trichogramma ostriniae*	Monoterpene	Limonene	Mung bean, *Vigna radiata*	[59]
Attractive (enhanced foraging)	*T. brassicae*, *T. evanescens*	Terpenes	(E)-β-ocimene, linalool	Black mustard, *Brassica nigra*	[60]
Attractive (enhanced foraging)	*T. achaeae*	Monoterpene, fatty acid ester, terpene ester	α-pinene, (Z)-3-hexenyl acetate, tetradeca-3,8,11-trienyl acetate	Tomato, *Solanum lycopersicum*	[9]
Attraction	*T. papilionis*	Aromatic ketone, sesquiterpenoid, ether, terpene, ester, phthalate ester, phenylpropene, phthalate	Acetophenone, anisole, α-farnesene, β-myrcene, cis-3-hexenyl butyric acid, bis(2-ethylhexyl) phthalate, trans-isoeugenol, Di-2-ethylhexylphathalate	Maize, *Zea mays* and sun hemp, *Crotalaria juncea*	[47]
Attraction	*T. dendrolimi*	Ester, aldehyde, terpene, ester	Methyl salicylate, benzaldehyde, linalool oxide, Cis-3-hexenyl caproate	Pear, *Pyrus communis* Peach, *Prunus persica*	[61]
Attraction	*T. bournieri*	Sesquiterpene hydrocarbon	(E)-(1R,9S)-caryophyllene	Maize, *Zea mays*	[62]
Attraction	*T. chilonis*	Phenol, ortho-cresol	2-phenylethanol, *o*-cresol	Tomato, *Solanum lycopersicum*	[63]
Attraction	*T. pretiosum*	Terpenoid, sesquiterpene, sesquiterpene aromatic hydrocarbon, monoterpene ketone, ester, sesquiterpene, and benzenoid/salicylate derivative	Linalool, β-gurjunene, calamene, thujone, (Z)-3-hexenyl acetate, (E,E)-α-farnesene, and methyl salicylate	Maize, *Zea mays*	[64]
Weak attraction of *T. brassicae*, *T. evanescens* to healthy plant odor (40–45%), whereas *Trichogramma embryophagum* was positively attracted to healthy plant odor (60%)	*T. brassicae*, *T. evanescens*, *T. embryophagum*	Not informed	Not informed	Tomato, *Solanum lycopersicum*	[65]
Positive chemotactic behavior	*T. pretiosum*	Jasmonate, phenolic acid	Methyl jasmonate, salicylic acid	Rice, *Oryza sativa*	[66]
Attraction	*T. japonicum*	Alkane, ester	Eicosane, octane, dodecane, tetrapentacontane, dotriacontane, henicosane, octadecane, ethyl acetate	Cow pea, *Vigna unguiculata*; green gram, *Vigna radiate*; black gram, *Vigna mungo*; soybean, *Glycine max*	[67]
Attraction	*T. pretiosum*	Jasmonate	Methyl jasmonate	Tomato, *Solanum lycopersicum*	[68]
Attraction	*T. brassicae*	Nitriles, alcohols, ketones, isothiocyanates, esters, terpenes, sesquiterpenes, disulfides, monoterpenes	2-butenenitrile, 3-butenenitrile, 1-penten-3-ol, 3-pentanone, 2-methylbutanenitrile, (Z)-3-hexen-1-ol, allyl isothiocyanate, (Z)-3-hexen-1-ol acetate, (E)-4,8-dimethyl-1,3,7-nonatriene (E-DMNT), 7-b-H-silphiperfol-5-ene, silphiperfol-6-ene, dimethyl disulphide, myrcene, isomenthone, 7-a-H-silphiperfol-5-ene, pre-silphiperfol-7-ene, silphiperfola-5,7(14)-diene, alpha-funebrene, longifolen, beta-caryophyllene, alpha-caryophyllene, (E,E)-alpha-farnesene, tricyclo[6.3.0.0(1,5)]undec-2-en-4-one, 2,3,5,9-tetramethyl (TUT),	Black mustard, *Brassica nigra*	[69]
Lower attraction	*T. achaeae*	Nitriles, isothiocyanates, alcohols	2-butenenitrile, allyl isothiocyanate, 3-pentanone, (Z)-3-hexen-1-ol	Resistant wild tomato, *Solanum arcanum*	[70]
Higer attraction	*T. achaeae*	Alcohols, terpenes, sesquiterpenes	(Z)-3-hexen-1-ol acetate, myrcene, alpha-caryophyllene, beta-caryophyllene	Resistant domesticated tomato, *Solanum neorickii*, *Corona F1*	
Higher attraction	*T. achaeae*	Nitriles, alcohols, terpenes	1-penten-3-ol, (E,E)-alpha-farnesene, (Z)-3-hexen-1-ol	Susceptible tomatoes, *Rentita, Romabelle F1*	
Attraction	*T. chilonis*	Monoterpenes, sesquiterpenes, aldehydes	Myrcene, phellandrene, caryophyllene, limonene, carene	Tomato, *Solanum lycopersicum*	[71]
Octadecane application significantly enhanced parasitoid attraction	*T. chilonis*	Saturated hydrocarbons	n-hexadecanoic acid, n-octadecanoic acid, octadecane	Wheat, *Triticum* spp. and chickpea, *Cicer arietinum*	[58]

**Table 2:** Use of *Trichogramma* species in biological pest control.

Authors	*Trichogramma* Species Used	Pest Species	Crop/Host Plant	Effectiveness in Pest Control	Geographical Distribution	Release Method	Number of Releases	Number of Wasps Per Release	Benefits	Challenges	Applicability (Crop/Area)
Gavara, Cabello [133]	*T. achaeae*, *T. euproctidis*	Tecia solanivora solanivora (Guatemalan potato moth)	Potato crops	High, particularly under field conditions	Canary Islands, Spain	Laboratory & semi-field tests	2 releases	60 wasps per release	Efficient in searching for eggs in soil, adaptable for field use	Limited performance under dark conditions; unsuitable under storage conditions	Potato crops in Canary Islands
Steidle, Rees [134]	*T. brassicae*, *T. pretiosum*, *T. carverae*	*Ephestia kuehniella* (Mediterranean flour moth or mill moth), *Ephestia cautella* (almond moth)	Stored products (grain)	Moderate, low host acceptance and fecundity	Australia	Inundative release	NI	NI	*T. brassicae*: Low host acceptance; inexpensive; *T. pretiosum*: Best for use, high fecundity and host acceptance; *T. carverae*: Good host-finding ability, low host acceptance	Low acceptance, especially at low host density	Stored grain-processing facilities
Murali-Baskaran, Chander Sharma [135]	*T. japonicum*	*Scirpophaga incertulas* (Yellow Stem Borer)	Rice (cv. Swarna)	Significant reduction in dead heart (54.7%) and white ear (66.1%)	India (Raipur, Chhattisgarh)	Inundative release	4 (weekly intervals)	50,000 wasps/ha × 4 releases	Effective in reducing pest damage and improving yield	Dependent on climate and pest cycles; requires careful timing of releases	Rice fields, particularly for controlling YSB
Yang, Li [136]	*T. chilonis*, *T. dendrolimi*, and *T. pretiosum*	*Spodoptera frugiperda* (Fall Armyworm)	Maize (corn)	Parasitism rates: *T. chilonis*: 10.65–24.49%; *T. dendrolimi*: 17.90–31.40%; *T. pretiosum*: 16.61–30.20%	China, Hong Kong	Inundative releases of parasitized egg cards (each containing approximately 2000 parasitized eggs). Release conditions: Parasitoid release ratio of 1:1 (parasitoids to FAW eggs).	Five releases	100 wasps were released per cage along with 100 FAW eggs	Significant reduction in damage rate and index and maize yield increase of 19.4%	Inconsistent parasitism across different species in terms of environmental conditions, particularly temperature.	Effective in Hainan, Guangdong, and Hong Kong, with potential for broader expansion.
Tang, Babendreier [137]	*T. japonicum*, *T. chilonis*	*Scirpophaga incertulas* (Yellow Stem Borer)	Rice (*Oryza sativa*)	In cage tests: *T. japonicum*: 60% parasitism, 15.8% egg parasitism. *T. chilonis*: 40.7% parasitism, 2.8% egg parasitism. In field trials: *T. japonicum*: 9.0% ± 7.6% parasitism, 0.35% ± 0.36% egg parasitism. *T. chilonis*: 15.1% ± 14.1% parasitism, 0.68% ± 0.66% egg parasitism	Southwestern China	Inundative release	Three release densities were tested: 50,000/ha, 100,000/ha, and 200,000/ha wasps.	50,000, 100,000, and 200,000 wasps per hectare, at 100 release points per hectare	*T. japonicum* showed higher parasitism than *T. chilonis* in both tests. *T. chilonis* was effective but had lower rates.	Low parasitism, especially for *T. chilonis* Egg mass accessibility hindered by protective hairs Concerns over mass-reared wasp quality due to transport/rearing conditions	Mainly for rice fields in regions affected by *Scirpophaga incertulas*, particularly in Southwestern China.
Sutil, Roswadoski [138]	*T. pretiosum*	*Spodoptera frugiperda* (Fall Armyworm), *Helicoverpa armigera* (Cotton bollworm)	Maize (corn)	*T. pretiosum* preferred *H. armigera* eggs (13.5%) over *S. frugiperda* (3.2%).	Brazil	*T. pretiosum* was released in envelopes at 100,000 parasitoids per hectare.	Three releases were performed per treatment over two consecutive maize seasons.	*T. pretiosum*: 100,000 parasitoids/ha	*T. pretiosum* is more effective on *H. armigera*.	*T. pretiosum* struggled with thick-shelled eggs	These parasitoids can control *S. frugiperda* and *H. armigera* in maize.
Xue, Tariq [139]	*T. leucaniae*	*Leguminivora glycinivorella* (Soybean Pod Borer)	Soybean	*T. leucaniae* improved parasitism from 43.33% to 90% over ten generations and was more effective on soybean pod borer eggs when reared on eri silkworm eggs.	China	Inundative release	Multi-generational rearing (F1 to F10) with regular parasitoid releases over successive generations	High-density releases	Eri silkworm eggs enabled cost-effective rearing of *T. leucaniae* with over 80% parasitism.	Rearing on *Corcyra* and *Antheraea* reduced performance, while eri silkworm is superior but needs adaptation.	Eri silkworm eggs are a sustainable source for mass-rearing *T. leucaniae*, boosting biocontrol in soybean crops.
Raven and Nahrung [140]	*T. carverae*, *T. pretiosum*, *T. nr finiculatum*	*Pericyma cruegeri* (Poinciana looper)	Poinciana (*Delonix regia*) trees	All *Trichogramma* species reduced *Pericyma cruegeri* emergence by 58%, with *T. nr finiculatum* most effective, averaging 2.1 wasps per egg.	Australia	Inundative release	Single release	NI	All *Trichogramma* species parasitized *P. cruegeri* eggs, with *T. nr finiculatum* being the most effective for pest control.	*T. carverae* was slower than *T. nr finiculatum*, and multiple wasp emergence may reduce efficiency.	Ideal for urban Queensland, offering a sustainable, non-chemical solution for Poinciana looper control.
Jiang, Zhou [97]	*T. ostriniae*, *T. chilonis*, *T. dendrolimi*	*Tuta absoluta* (Tomato Leafminer)	Tomato	*T. ostriniae* outperformed *T. chilonis* and *T. dendrolimi* in parasitizing *Tuta absoluta* eggs, with better performance at all ages.	China	Inundative release	Multiple release sessions	One female *Trichogramma* was released per dish with 30 *Tuta absoluta* eggs.	*T. ostriniae* was most effective, then *T. chilonis*, with *T. dendrolimi* least effective on older eggs.	As egg age increased, parasitism and emergence decreased. *T. dendrolimi* struggled with older eggs, reducing its field reliability.	*T. ostriniae* is best for *Tuta absoluta* control in Yunnan tomatoes, with *T. chilonis* as a secondary option.
Myint, Huang [141]	T. ostriniae, T. dendrolimi	*Ostrinia furnacalis* (Asian corn borer)	Maize	*T. ostriniae* from Yatsawk had 89% parasitism, increased maize yield by 60%, and reduced damage and yield loss by 50%.	Myanmar	Inundative releases	Two releases	Three release densities were tested: 50,000 wasps/ha (low), 100,000 wasps/ha (medium), 200,000 wasps/ha (high).	*T. ostriniae* from Yatsawk reduced plant damage (60–80%) and yield loss (50%), boosting maize yield by 60%.	Optimal release density, timing, and environmental factors are crucial for pest control, with field validation needed for long-term efficacy.	This study provides a sustainable, cost-effective alternative to pesticides for maize in Myanmar.
Wang, He [142]	*T. dendrolimi*, *T. chilonis*, *T. ostriniae*	*Ostrinia furnacalis* (Asian corn borer), *Helicoverpa armigera* (Cotton bollworm), *Conogethes punctiferalis* (Yellow peach moth)	Corn (*Zea mays*)	*T. ostriniae* controls *Ostrinia furnacalis* in China, parasitizing 90% of eggs, while *T. dendrolimi* and *T. chilonis* offer variable effectiveness.	Northeast China, Southwest China	Inundative release	Releases occurred in two Asian corn borer generations, with 150,000–300,000 wasps per hectare and multiple releases per season.	150,000 to 300,000 wasps per hectare	*Trichogramma* reduced damage by 92%, increased parasitism to 85%, and reduced pesticide use.	*Trichogramma* effectiveness varies by climate, and production is costly with storage and transport challenges.	*Trichogramma* is used in key corn regions to control *O. furnacalis*, *H. armigera*, and *C. punctiferalis*.

NI: not informed.

## Data Availability

No new data were created or analyzed in this study. Data sharing is not applicable to this article.
